# The Shear Performance of an Aircraft off Pin Made of Quartz Fiber Reinforced Phenolics: The Effect of the Fiber Distribution Property

**DOI:** 10.3390/ma17225483

**Published:** 2024-11-10

**Authors:** Ziwei Huang, Jianwei Ren, Yan Xia, Zhenyu Zhao

**Affiliations:** 1 State Key Laboratory of Mechanics and Control for Aerospace Structures, Nanjing University of Aeronautics and Astronautics, Nanjing 210016, China; vv.hunag@nuaa.edu.cn; 2 MIIT Key Laboratory of Multifunctional Lightweight Materials and Structures, Nanjing University of Aeronautics and Astronautics, Nanjing 210016, China; 3 High-Tech Institute of Xi’an, Xi’an 710025, China; jewaren0621@nuaa.edu.cn; 4 China Academy of Launch Vehicle Technology, Beijing 100076, China; jane__xia@163.com

**Keywords:** fibrous composite material, fiber distribution property, aircraft breakaway pin, double-sided shear test

## Abstract

Emergency breakaway pins (EBPs) have been widely used in aircraft, especially in the suspension connection between the engine device and the airfoil. Currently, the existing EBPs, which are made of metal materials, barely satisfy the lightweight requirement of the airplane industry. Thus, the construction of a novel EBP with quartz fiber reinforced phenolics is proposed in this study, and the shear response is examined experimentally using a double-sided shear test. The effect of the fiber distribution characteristic on the shear strength is then assessed quantitatively. The failure patterns, including the damage morphology of the two types of samples were then reconstructed using scanning electron microscopy (SEM). Experimental results showed that the breakaway composite pin fabricated by the laminated composite had a superior shear response than its counterpart with randomly distributed fibers for its uniaxially distributed fiber yarns provided a longer put-out damage trace that contributed to a higher shear-loading bearing capacity for the entire composite EBP. In specific, the average values of the shear strength and the shear stiffness for the former samples were higher by 61% and 22%, respectively, than that for the latter samples. Additionally, the composite EBP also has an excellent combination of lightweight advantage and stronger shear-bearing capacity over competing pins, providing novel insight for more secure designs for civil and military aviation.

## 1. Introduction

**Figure 1:**
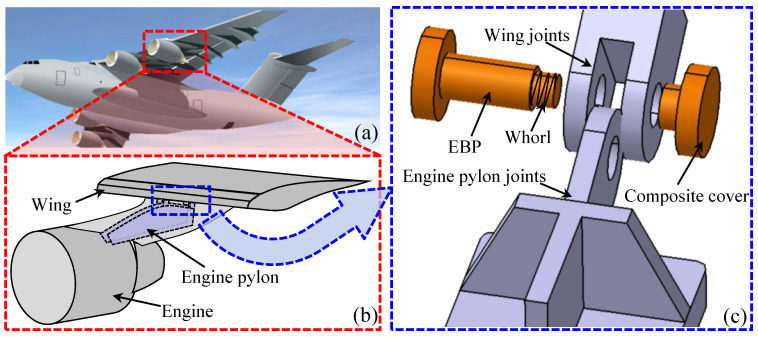
(**a**) A civilian airliner. (**b**) Partial view of the engine and wing connection. (**c**) Connection mechanism of the engine and wing.

An emergency breakaway pin (EBP) is an anti-overloading element between the hanging device and the airfoil, and it has been widely applied in civil aircraft pylons [1] and space pyrotechnic devices [2]. Under an emergency or overloading condition, the EBP breaks automatically to unlock the hanging devices to avoid overloading. The use of such a component can prevent a security threat to an aircraft and its occupants against in-air accidental airfoil damage caused by a puncture to the engine pylon, as well as the resulting explosion that could be caused by a tear rupture of the overloaded fuel tank. For example, Figure 1 shows an EBP application in a civil aircraft pylon, and Figure 1a,b shows the details of an aircraft pylon system between the engine device and the airfoil [3]. Figure 1c is a schematic of the connecting mechanism of an EBP element. Under such a scenario, an EBP element is a critical and important load-bearing component in an aircraft pylon system. Thus, an exploration of the mechanical response of the EBP component, especially during shearing performance, may provide better insight into aviation security.

Currently, most EBP elements, which are composed of high-strength metals, have a high mass density [4]. This makes it difficult to meet the lightweight requirement of the aviation industry [5]. Thus, the design of an EBP element that balances mechanical performance and lightweight is urgently required. The mechanical natures of composite materials, such as carbon fiber reinforced nanocomposites [6], woven glass fiber composites [7], and quartz fiber reinforced composites with matrices of phenolic aerogels [8], can assist in such an endeavor. These composite materials are used widely in engineering [9] due to their high strength, stiffness, and thermal mitigation. In particular, a quartz fiber reinforced composite that consists of a reinforcing component made from high-purity quartz fiber offers a relatively higher strength and excellent thermal resistance. In addition, a quartz fiber reinforced composite is lightweight [10,11], which makes it more promising and advantageous than its counterparts. Considering these mechanical properties, quartz fiber reinforced composite materials may be a promising material candidate for the design of EBP elements.

In this study, the shear response of EBP samples made of quartz fiber reinforced composite materials is examined experimentally using a quasi-static double-sided shear test, with a further exploration of the effect of the fibers’ spatial distribution characteristics on the shear strength of the EBP. Two spatial distribution modes of the short-chopped fibers were considered in the EBP samples, i.e., a composite EBP containing uniaxially distributed fiber yarns and one containing randomly distributed fiber filaments. After the shear test, failure morphologies of each type of EBP sample were captured using a scanning electron microscopy (SEM), and the shear strength of each scenario was assessed quantitatively. Additionally, the underlying mechanism of the distribution property of the fiber yarns on the EBP mechanical performance was elucidated in detail. Finally, a comparison of the composite EBP samples proposed here with the existing counterparts was conducted. The EBP composed of quartz fiber reinforced phenolics exhibited an excellent combination of shear-bearing capacity and lightweight advantages than the existing EBPs, providing novel insight for more secure designs for civil and military aviation.

## 2. Materials, Sample Fabrication, and Experimental Procedures

**Figure 2:**
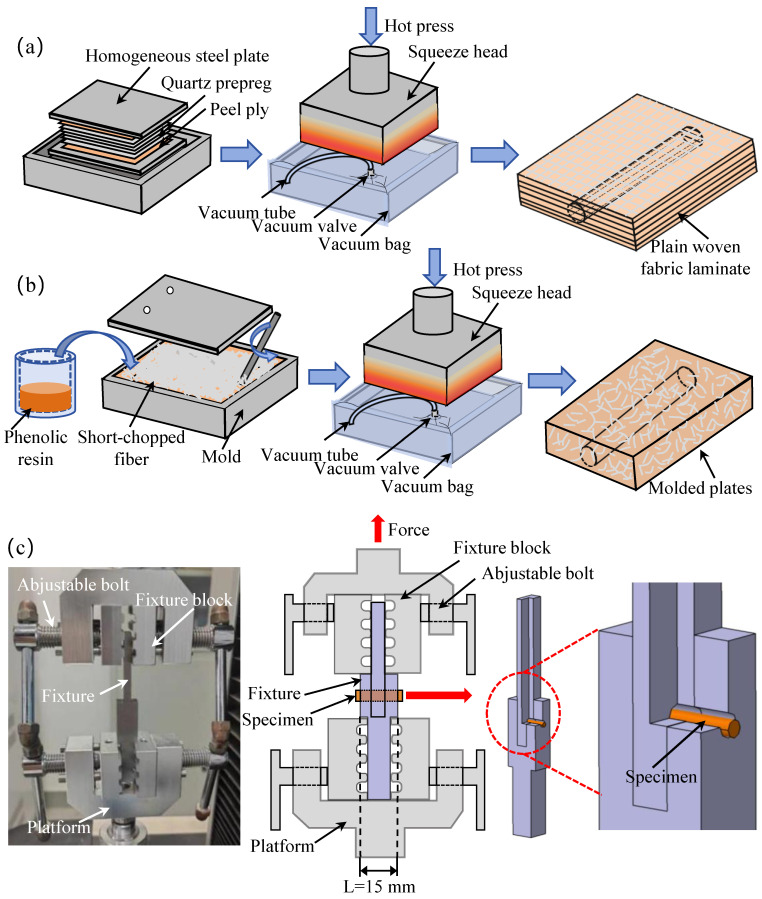
(**a**) Preparation processes of LP specimen [15]. (**b**) Preparation processes of MP specimen. (**c**) Test setup used for double-sided shear test.

The silica-magnesium phenolic premix (acquired from Tongshan Co., Ltd., Tangshan, China) consisted of 40% phenolic resin. The composite fabric (supplied by Huatek New Material Inc., Xianyang, China) was weaved automatically by the quartz fibers with a diameter of 7 μm and a mean thickness of 0.25 mm. All EBP samples were prepared using the hot-consolidation manufacturing process. Two corresponding sample manufacturing approaches were adopted to obtain two types of samples. For the EBP samples with orderly distributed fibers, composite stripes containing the quartz fibers were stacked alternatively in a 0°/90° consequence [12,13]. The laminated prepreg panel was then consolidated in a hot press using a preheated steel die with a pressure of 3 MPa and temperature of 150 °C for 180 min. The die/sample assembly was then cooled to ambient temperature (20 °C). Subsequently, the bar-shaped EBP sample with a diameter of 2.5 mm was machined via a water-jet machine (Figure 2a). A similar manufacturing process was also used to prepare the EBP samples containing randomly distributed fibers. However, it is emphasized that the composite panel was fabricated using the modeled press procedure. In this process, the short-chopped fiber having an average length of 2 mm was first manufactured. These short-chopped fibers were then positioned randomly within the steel die system. Subsequently, a phenolic resin was poured into the mold, and the compound consisting of phenolic resin and short-chopped fibers was stirred until the chopped fibers were completely uniform in the hybrid fluid-like preform [14]. The mold and composite preform system was then packaged together with vacuum bags and vacuum prepressed to consolidate this preform component. After the heating–cooling process, EBP samples with randomly distributed shot-chopped fibers were obtained (Figure 2b). To provide a clear distinction, the EBP samples with uniaxially and randomly distributed fibers were labeled as LP and MP, respectively.

With reference to the testing standard of the International Organization for Standardization (ISO) 8749:1986 [16], double-sided shear tests were performed using a universal testing machine (WH-70, Weiheng Testing Machine Co., Ltd., Ningbo, China). To measure the shear response of the EBP samples, a fixture/sample system was purposely designed, as shown in Figure 2c. This sample’s fixture included a loading yoke accompanied with a loading pin along the axial direction. Through-thickness holes were positioned in both the yoke and pin components along the transverse direction. Additionally, two fixture blocks that had jagged edges were used to clamp both ends of the fixture system mentioned above. The EBP samples were inserted into the through-thickness holes of the fixtures, and then the fixture was clamped on the platform of the universal testing machine. Subsequently, the out-of-plane shear response of the EBP samples was imposed and then measured experimentally by stretching the upper end of the fixture (Figure 2c). In this study, a load cell with a measuring range of 1000 N–5000 N applied a quasi-static load with a loading rate of 0.5 mm/min. Once damage to the sample occurred, the loading test was stopped, and the measurement data were recorded.

The aim of this shear test was to quantitatively investigate the effect of the fiber’s distribution on the shear strength of the composite EBP. Two types of composite EBP samples were examined, and three repeated tests were conducted to delineate the experimental error. All of the experimental measurements are reported here, including the geometrical sizes and the extreme shearing strengths that were obtained by computing the corresponding average value of each group. Subsequently, the detailed damage morphologies of each scenario were characterized using scanning electron microscopy (SEM) (Phenom XL, Amsterdam, Netherlands) to elucidate the underlying damage mechanism of such a composite EBP.

## 3. Results and Discussion

### 3.1. Shear Response of Double-Sided Shear Test and Microscopic Characterization

**Figure 3:**
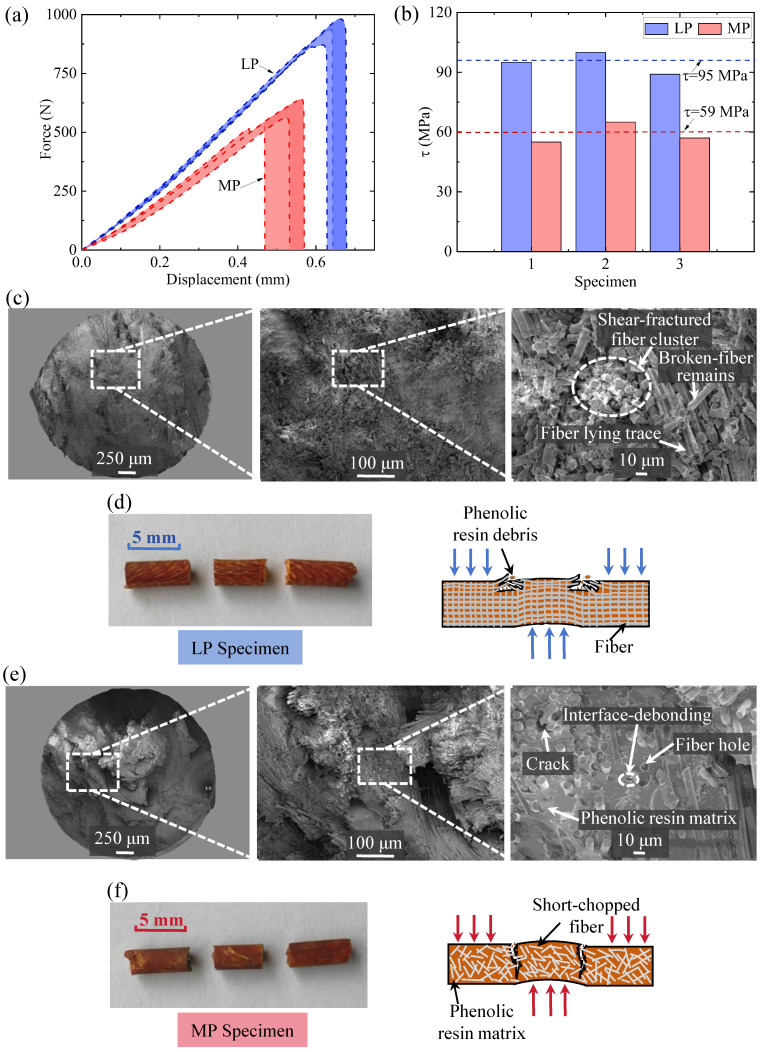
(**a**) Load versus displacement of LP and MP pin specimens. (**b**) Shear strength column diagram of each specimen. (**c**) SEM image of LP specimen. (**d**) Double shear failure schematic diagram of LP specimen. (**e**) SEM image of MP specimen. (**f**) Double shear failure schematic diagram of MP specimen.

The measured load–displacement curves of each EBP sample were plotted and shown in Figure 3a. The imposed shear force increased with increasing displacement until damage occurred. Once the loading section began to be damaged, the force decreased sharply. The comparison shown in Figure 3a indicates that all of the LP EBP samples had superior shear performances, including higher shear strengths and larger failure displacements. Correspondingly, these plotted curves of force versus displacement also show that the uniaxial distribution of fibers contributed to an enhancement in the shear stiffness of the composite EBP samples, i.e., the higher ratio of shear force to the displacement. The average values of the failure displacement and the shear stiffness for the LP samples were higher by 24% and 22%, respectively, than that for the MP samples. In specific, Figure 3b shows the shear strength of each sample, including the MP and LP cases, for a clear comparison. This comparison showed that the average shear strength of the LP samples was 95 MPa, which was 1.61 times that of the MP samples. Additionally, Table 1 shows the fracture displacement, stiffness, and shear strength of each sample, and provides the analysis for the deviation of the corresponding average values, respectively. Based on these measurements, we concluded that the uniaxial distribution of the continuous fibers contained within the composite EBP component significantly reinforced the shear resistance of the entire pin component.

Furthermore, a characterization of the typical damage morphologies of the two EBP samples was conducted to rationalize the underlying mechanism of the spatial fiber distribution. Figure 3c,d is the SEM images and the pictures of the postmortem LP sample. It can be seen from Figure 3c that the fibers were distributed orderly in the shear damage section. In addition, several fiber yarns caused the damage pattern induced by shear, and this was accompanied by a pull-out failure mode. For most of the fiber clusters, there was a good adhesion condition between the reinforcing fiber and the phenolic resin matrix, and most of the fibers were coated evenly by the resin matrix. The visible damage characteristics were consistent with those mentioned in a previous report [17]. Also, the visualized fiber lying trace can be seen in the right sub-figure of Figure 3c marked by a hollow circular line. These detailed damage morphologies revealed that the damage process of the LP sample primarily consisted of the following two stages: (i) the debonding between the fiber and matrix and (ii) the pull-out damage of the broken fiber from the matrix. For better understanding, a picture of the post-damage LP sample and its schematic showing the damage mechanism is shown in Figure 3d. In contrast, the SEM images of the MP samples are shown in Figure 3e. For the MP sample, the broken fibers at the damage section are disordered, accompanied by a randomly distributed crack. In addition, the typical damage characteristics also included the following: (i) a relatively flatter failure section and (ii) numerous voids induced by the removal of fiber. From these observations, it can be inferred that the damage to all of the MP samples was initiated from a crack that developed from the tiny debonding crevices around the broken fibers [18]. To better visualize this, Figure 3f provides pictures of the post-tested MP sample and an illustration of its damage mechanism.

Based on the visualized damage morphologies of the tested LP and MP samples, the effect of fiber distribution on the shear response of the entire EBP sample could be explained. For the LP samples, many of the continuous fiber yarns were positioned along the longitudinal direction. When the shear loading was applied, the continuous fiber suffered (i) a stress localization and a subsequent fracture and (ii) a pull-out failure with a longer pull-out trace. During the damage process, the continuous fibers provided connection bridges between the broken matrix portions that resulted in the fibers around the damage section being subjected to a larger load [19,20]. Under such a scenario, the shear strength of the entire EBP sample was governed simultaneously by the strength of the fiber and matrix, and the damage characteristic was dominated by the failure mechanism that competed for the relevant two components. In contrast, in the MP sample, there were fewer connecting bridges that were provided by the short-chopped fiber. This meant that the EBP sample would fail as soon as the interface-debonding crevices initiated and subsequently developed into a through-section crack. In this case, the shear strength was strongly governed by the strength of the matrix and only weakly affected by the fiber.

### 3.2. A Comparison of the Shear Strengths of the Competing EBPs

**Figure 4:**
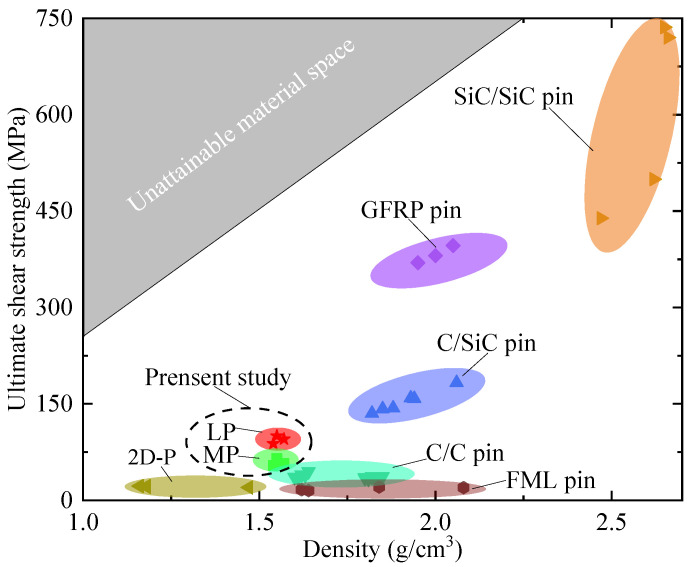
Comparison of lightweights of currently available counterparts [21,22,23,24,25,26,27].

A comparison of the composite EBP samples proposed here with the existing counterparts was conducted, and the comparison chart is shown in Figure 4. In this comparison, the promising combinations of lower density and ultimate shear strength of each EBP were also considered. Except for the LP and MP samples, the compared EBP targets also included EBPs constructed with the carbon/silicon carbon (C/SiC) composite [21], the carbon/carbon (C/C) composite [22,23], the GFRP composite [24], the silicon carbon/silicon carbon (SiC/SiC) composite [25], the 2D-P composite [26], and the FML composite [27]. The comparison figure shows that the composite EBP proposed here delivered a comparatively higher shear strength in the lightweight range. This means that the EBP composed of quartz fiber reinforced phenolics exhibited an excellent combination of shear-bearing capacity and lightweight advantages than the existing EBPs.

## 4. Conclusions

This study used a double-sided shear test to experimentally investigate the effect of the fiber spatial distribution on the shear response of a composite EBP made of quartz fiber reinforced phenolics. The detailed damage modes of the two types of samples prepared using different manufacturing processes were characterized, and the damage mechanism of each EBP sample was investigated. Results showed that the EBP sample that contained uniaxially distributed fibers had a stronger shear-bearing capacity that was 1.61 times higher than that of the sample that contained randomly short-chopped fibers. This was because the uniaxially distributed fibers provided a high load-bearing capacity and a longer put-out damage trace that contributed to a higher shear-loading bearing capacity for the entire composite EBP. Furthermore, the EBPs proposed here produced a superior combination of a stronger shear-bearing capacity and a lightweight property. However, the double-sided shear test of the breakaway pin studied in the paper could not represent the application of EBP entirely. In order to provide more secure designs for civil and military aviation, the mechanical response of the break-away pin will be analyzed under thermodynamic coupling conditions through laboratory experiments and numerical simulations in future research.

## Figures and Tables

**Table 1:** Error in each test case of EBP.

Specimen Type	Number	Fracture Displacement (mm)	Error	Stiffness (N/mm)	Error	Shear Strength (MPa)	Error
LP	No. 1	0.64	0.000	1426.7	−16.4	95	+0.3
	No. 2	0.66	+0.020	1493.3	+50.2	100	+5.3
	No. 3	0.62	−0.020	1409.3	−33.8	89	−5.6
MP	No. 1	0.47	−0.046	1108.2	−70.5	55	−4.0
	No. 2	0.56	+0.043	1253.9	+75.1	65	+6.0
	No. 3	0.52	+0.003	1174.1	−4.6	57	−2.0

## Data Availability

Data are contained within the article.
